# Evaluation of Healthcare Utilisation and Expenditures in Persons with Type 2 Diabetes Undergoing Bariatric-Metabolic Surgery

**DOI:** 10.1007/s11695-023-06849-z

**Published:** 2024-01-10

**Authors:** Valerie M. Monpellier, Rose J. Geurten, Ignace M.C. Janssen, Dirk Ruwaard, Jeroen N. Struijs, Peter R. van Dijk, Henk J.G. Bilo, Arianne M.J. Elissen

**Affiliations:** 1 Nederlandse Obesitas Kliniek (Dutch Obesity Clinic), Huis ter Heide, The Netherlands; 2 Department of Health Services Research, CAPHRI Care and Public Health Research Institute, Faculty of Health, Medicine and Life Sciences, Maastricht University, Maastricht, The Netherlands; 3 Department of Quality of Care and Health Economics, Centre for Nutrition, Prevention and Health Services, National Institute of Public Health and the Environment (RIVM), Bilthoven, The Netherlands; 4 Department Public Health and Primary Care, Leiden University Medical Centre, Campus The Hague, The Hague, The Netherlands; 5 Department of Endocrinology, University Medical Centre Groningen, University of Groningen, Groningen, The Netherlands; 6 Department of Internal Medicine, University Medical Centre Groningen, University of Groningen, Groningen, The Netherlands

**Keywords:** Type 2 diabetes mellitus, Bariatric-metabolic surgery, Healthcare expenditures, Healthcare costs, Healthcare utilisation, Medication

## Abstract

**Purpose:**

Changes in healthcare utilisation and expenditures after bariatric-metabolic surgery (BMS) for people with type 2 diabetes mellitus (T2DM) are unclear. We used the Dutch national all-payer claims database (APCD) to evaluate utilisation and expenditures in people with T2DM who underwent BMS.

**Methods:**

In this cohort study, patients with T2DM who had BMS in 2016 were identified in the APCD. This group was matched 1:2 to a control group with T2DM who did not undergo BMS based on age, gender and healthcare expenditures. Data on healthcare expenditures and utilisation were collected for 2013–2019.

**Results:**

In total, 1751 patients were included in the surgery group and 3502 in the control group. After BMS, total median expenditures in the surgery group stabilised (€ 3156 to € 3120) and increased in the control group (€ 3174 to € 3434). Total pharmaceutical expenditures decreased 28% in the surgery group (€957 to €494) and increased 55% in the control group (€605 to €936). In the surgery group, 67.1% did not use medication for T2DM in 2019 compared to 13.3% in the control group. Healthcare use for microvascular complications increased in the control group, but not in the surgery group.

**Conclusion:**

BMS in people with T2DM stabilises healthcare expenditures and decreases medication use and care use for microvascular complications. In contrast, healthcare use and expenditures in T2DM patients who do not undergo surgery gradually increase over time. Due to the progressive nature of T2DM, it is expected that these differences will become larger in the long-term.

**Graphical Abstract:**

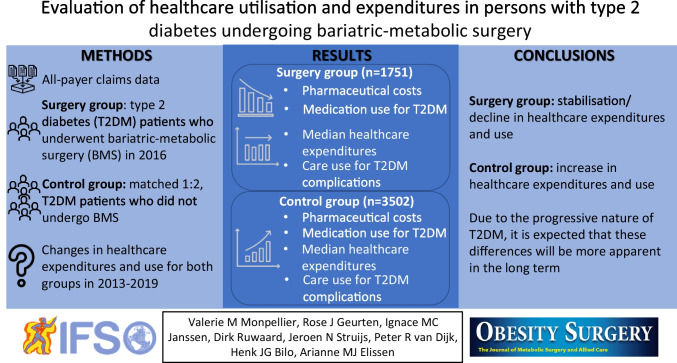

**Supplementary Information:**

The online version contains supplementary material available at 10.1007/s11695-023-06849-z.

## Introduction

Obesity and type 2 diabetes mellitus (T2DM) often coincide and have a major impact on patients’ health and wellbeing [1–3]. To date, bariatric-metabolic surgery (BMS) remains the most effective treatment for patients with obesity and T2DM with regard to health outcomes. In the STAMPEDE trial, T2DM patients with a body mass index (BMI) between 27 and 43 kg/m^2^ were randomised to lifestyle treatment or BMS, with outcomes assessed during a 5-year follow-up period [4]. Patients who underwent surgical treatment had lower glycated haemoglobin and fasting plasma glucose levels for the complete follow-up period. Similarly, prior research has shown positive effects of BMS on diabetes-related cardiovascular complications as well as renal, ophthalmic and neurological outcomes [5–10]. However, the impact of BMS for people with T2DM in terms of healthcare utilisation and expenditures has not been studied in depth.

Treatment of obesity with BMS is costly, with prices ranging from around €6047 in New Zealand, €7800 in the Netherlands and €26,000 in the USA for the procedure alone [11–13]. Because of these relatively high costs, many studies have assessed the cost-effectiveness of BMS: recent meta-analyses concluded that BMS for people with T2DM is cost-effective [14] or even cost-saving [15]. However, the majority of included studies in these analyses used estimates of costs and models to evaluate changes in costs. Although these models are a well-known and validated method to evaluate cost-effectiveness, data on the real change in healthcare expenditures or utilisation that occurs after BMS for people with T2DM are lacking or come from small-sized studies. In addition, analyses rarely include complications of BMS, additional costs that arise after surgery (e.g. body contouring procedures), common associated medical problems of obesity and healthcare expenditures and utilisation for diabetes-related complications [14–16].

Thus, while BMS is an effective treatment for obesity in T2DM in terms of medical outcomes and research suggests that cost savings are possible, real-world evidence on associated changes in healthcare expenditures and utilisation is still largely missing. In this study, we present a first exploration of these changes based on nation-wide data from an all-payer claims database in the Netherlands. The study focuses on changes in total healthcare expenditures and utilisation, specific subtypes of healthcare (e.g. primary care, pharmaceutical) and care for diabetes-related complications. In addition, we provide a first exploratory comparison with a group of people with T2DM who did not undergo BMS.

## Methods

### Study Design

This is a retrospective, matched cohort study. Nation-wide healthcare expenditure and utilisation data were drawn from the Vektis Healthcare Information Centre in the Netherlands, which manages the all-payer claims database (APCD) that contains reimbursement data of all Dutch citizens (99% coverage) [17]. Since BMS is reimbursed care for adults in the Netherlands, all performed procedures are available in this database.

Data were retrieved from the APCD in April 2021 based on a detailed data extraction and processing request (see patient selection). In compliance with privacy laws, the researchers received aggregated data (i.e. only data of groups and not individual patients was available) which is not traceable to individuals. According to the Maastricht University Medical Centre ethics committee, this study was therefore not subjected to the Dutch ‘Research involving Human Subjects act’ (registration number: 2021-2591).

The STROBE guideline was used for this manuscript.

### Selection of Study Population

**Fig. 1:**
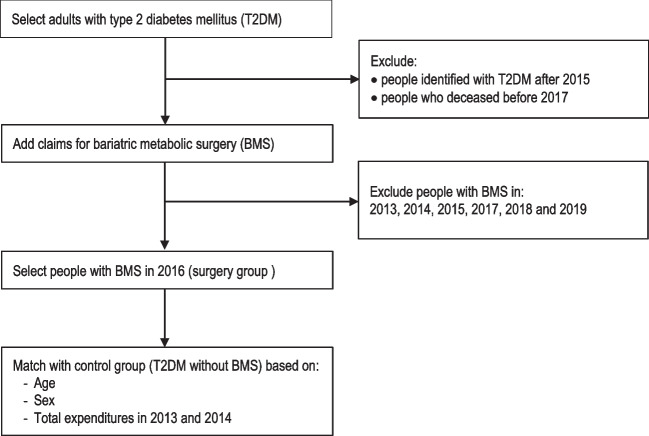
Procedure for selection of the study population from the Dutch all-payer claims database

Recently, we developed a method to select persons with T2DM based on their claims data as included in the Dutch APCD [18]. For the current study, this selection process was adjusted to identify a ‘surgery group’ of people with T2DM who underwent BMS in 2016 and a matched control group (1:2 ratio) of people with T2DM who did not undergo BMS (Fig. 1). The year 2016 was chosen to ensure that claims data were available in the APCD 3 years prior to BMS and 3 years after BMS.

#### Step 1: Selecting People with Type 2 Diabetes Mellitus

People with T2DM were identified based on claim codes showing use of integrated diabetes care and/or medication use for type 2 diabetes (i.e. oral blood glucose lowering medication, insulin or a combination medication: insulin/GLP-1) in 2015 or before [18].

People who deceased before 2016 were excluded. In addition, people who underwent BMS in 2013, 2014, 2015, 2017, 2018 or 2019 were excluded in order to determine the surgery and control group in steps 2 and 3.

#### Step 2: Selection of People Who Had Undergone Bariatric-Metabolic Surgery

People who underwent BMS in 2016 (surgery group) were selected from the T2DM population based on surgery reimbursement claims.

#### Step 3: Selection of People Who Had Not Undergone Bariatric-Metabolic Surgery

Subsequently, a group of people with T2DM who had not undergone BMS during the study period (the control group) was matched on healthcare expenditures, age and sex in a 1:2 ratio to the surgery group (people with T2DM who had undergone BMS in 2016). Since the APCD does not include clinical parameters, total healthcare expenditures in the first 2 years before surgery (2013 and 2014) were used as a proxy of general health. First, total healthcare expenditures over these years were calculated for the total population of people with T2DM in the APCD. Second, this population was divided in quartiles based on their total costs. Third, an equal number of people who underwent BMS was selected from each quartile. These people were then matched to people who did not undergo BMS in the same cost quartile.

### Outcome Measurements

The APCD contains data on all healthcare activities covered by the Dutch Insurance Act, covering primary and secondary care. In this study, total healthcare use and expenditures as well as use and expenditures in primary care, secondary care (diabetes-related complications), mental healthcare and pharmaceutical care were considered. We did not include costs for institutionalised care nor for supportive care at home (for example, support for informal care persons).

Total healthcare utilisation and expenditures were based on all healthcare activities across healthcare sectors and service types. Primary care includes all care provided in the primary care practice, by either the general practitioners or practice nurses. Secondary care includes all care that is provided by medical specialists (e.g. surgeons, cardiologists, dermatologists). In the Netherlands, secondary care is reimbursed using ‘diagnosis-treatment combinations’ (DTCs). These DTCs were used to study secondary care and complications of T2DM [18]. For diabetes-related complications, healthcare use and spending for macrovascular and microvascular complications were considered. Macrovascular complications were defined based on DTC claims for heart failure, cerebrovascular and cardiovascular events. Microvascular complications were based on DTC claims for diabetic mono/polyneuropathy, diabetic renal disease, diabetic eye complications and peripheral diabetic angiopathy.

Mental healthcare includes basic and specialised mental healthcare.

Total use and expenditures of pharmaceutical care as well as reimbursements of medication for diabetes and associated medical conditions were retrieved from the APCD. For associated medical conditions, the following ATC codes were selected: antihypertensives (C02), diuretics (C03), beta blocking agents (C07), calcium channel blockers (C08), agents acting on the renin-angiotensin system (C09), lipid-modifying agents (C10).

### Statistical Analyses

Data were analysed descriptively; all data were presented for the surgery group and control group separately. Binary variables (sex, mortality rates, healthcare utilisation, medication utilisation) were presented as percentages of the population in the year analysed. Age was presented with mean ± standard deviation. Expenditures for total healthcare were presented as total expenditures of the whole population and median expenditures per patient per year. Expenditures for healthcare sectors (primary care, mental healthcare and secondary care) were presented as median expenditures per patient per year. Pharmaceutical expenditures were presented as median expenditures of all medication (including medication for diabetes associated medical conditions) and expenditures of diabetes medication per patient per year. For type 2 diabetes-related complications median expenditures were presented, as well as 5th percentile (p5) and 95th percentile (p95) of expenditures. For all reported expenditures, median healthcare expenditures per patient represent the median expenditures for the people who utilise the specific type of care.

## Results

### Study Population

In total, 1751 people with T2DM who underwent BMS in 2016 were included in the surgery group, and 3502 people were included in the control group. In both groups, the average age was 52 ± 9 years and 65% was female. Mortality in the years 2016–2019 was 1.3% (23/1751) in the surgery group and 2.3% (83/3502) in the control group.

### Total Healthcare Utilisation and Expenditures

Nearly 100% of the people in both groups used healthcare services in each year of the studied period (Table 1). For the surgery group, median expenditures per patient were highest in the surgery year (2016): €13,070. The year before surgery (2015) the median expenditures of the surgery group increased 43% compared to 2013 (€4526 versus €3156). After 2016, median expenditures in the surgical group decreased to €3120 in 2019, which was −1.1% compared to expenditures in 2013 (€3156). In the control group, expenditures increased to a median of €3434 in 2019, which was 8% higher compared to the expenditures in 2013 (€3,174).

**Table 1:** Overview of annual healthcareutilisation (% of group), median expenditures per person (of the people using healthcare), and total healthcare expenditures,  for the surgery and control group

*Year*	Surgery group (*N*=1751)			Control group (*N*=3502)		
	Utilisation	Median expenditures	Total expenditures	Utilisation	Median expenditures	Total expenditures
*2013*	99.7	€ 3156	€ 9,529,918	99.5	€ 3174	€ 22,330,291
*2014*	99.8	€ 3592	€ 10,760,613	99.9	€ 3311	€ 22,885,705
*2015*	100	€ 4526	€ 12,649,843	99.9	€ 2784	€ 20,727,222
*2016*	100	€ 13,070	€ 26,359,278	100	€ 3033	€ 22,862,161
*2017*	99.9	€ 3616	€ 11,895,381	99.6	€ 2990	€ 23,535,408
*2018*	99.8	€ 3119	€ 11,019,142	99.4	€ 3219	€ 24,148,916
*2019*	99.2	€ 3120	€ 11,234,337	99.1	€ 3434	€ 24,846,936

### Healthcare Categories

For the surgery group, primary care utilisation was highest in 2015, and expenditures were highest in 2016 (Table 2). After 2016, the primary care expenditures in the surgery group decreased from €504 in 2016 to €433 per patient per year in 2019. In the control group expenditures were highest in 2019 (€ 492). In both groups, primary care expenditures increased after 2015, because care for chronic conditions was then shifted to primary healthcare.

**Table 2:** Annual utilisation (% of group) and median expenditures (per person) per healthcare sector, for the surgurey and control group

*Year*	Primary care				Mental health care				Secondary care			
	Surgery group		Control group		Surgery group		Control group		Surgery group		Control group	
	Utilisation	Expenditures	Utilisation	Expenditures	Utilisation	Expenditures	Utilisation	Expenditures	Utilisation	Expenditures	Utilisation	Expenditures
*2013*	99.7	€ 164	99.3	€ 154	15.2	€ 1609	15.9	€ 2153	93.5	€ 1063	92.8	€ 990
*2014*	99.8	€ 181	99.6	€ 168	14.0	€ 2134	14.8	€ 2299	95.7	€ 1144	94.9	€ 979
*2015*	100.0	€ 489	99.7	€ 480	13.3	€ 1660	13.4	€ 2364	98.5	€ 1720	95.7	€ 685
*2016*	99.9	€ 504	99.8	€ 491	10.6	€ 1224	13.2	€ 2367	99.9	€ 10,820	95.8	€ 816
*2017*	99.9	€ 460	99.4	€ 487	11.4	€ 2160	11.9	€ 2390	99.0	€ 1886	94.9	€ 843
*2018*	99.8	€ 432	99.1	€ 482	11.7	€ 2172	11.8	€ 2463	97.1	€ 1468	95.4	€ 884
*2019*	99.1	€ 433	98.5	€ 492	11.4	€ 2243	10.0	€ 2372	95.1	€ 1375	94.6	€ 932

Mental healthcare utilisation in the surgery group was lowest (10.6%) in the surgery year. After that, there was an increase to 11.4%, but never as high as before surgery (15.2%). Median expenditures per patient for mental healthcare in 2016 were 76% of the expenditures 2013 (€1224 versus €1609) in the surgery group, but then increased to €2243 in 2019. Mental healthcare utilisation was comparable in the control group; however, the median expenditures in this group were higher in all study years.

Expenditures for secondary care were highest in the surgery year (€10,820 per patient). After 2016, both percentage of persons using secondary care and subsequent expenditures decreased to 95.1% and €1375, respectively, in 2019. In the control group, secondary care utilisation and expenditures were always lower compared to the surgery group.

### Pharmaceutical Care

In the surgery group, total medication utilisation was lowest in 2019 (97.2%) and highest in the surgery year (2016; 100%) (Table 3). After the surgery year, median expenditures decreased in the surgery group from €957 in to €494 in 2019 (−28%), whereas median per patient expenditures in the control group increased consistently in each year of the study period (from €605 in 2013 to €936 in 2019; + 55%).

**Table 3:** Annual utilisation (% of group) and median expenditures per person for any type of medication (total) and median expenditures per person per diabetes medication type, for the surgery and control group

	Surgery group (1,751)						Control group (*n* = 3,502)					
	Total medication^a^		Diabetes medication^b^				Total medication^a^		Diabetes medication^b^			
*Year*	Utilisation	Expenditures	Expenditures				Utilisation	Expenditures	Expenditures			
			Oral	Insulin	Oral/insulin	Other			Oral	Insulin	Oral/insulin	Other
*2013*	98.2	€ 686	€ 22	€ 822	€ 886	€ 0	97.2	€ 605	€ 17	€ 590	€ 648	€ 0
*2014*	99.0	€ 800	€ 23	€ 695	€ 961	€ 0	98.5	€ 649	€ 18	€ 468	€ 654	€ 0
*2015*	99.7	€ 957	€ 26	€ 947	€ 986	€ 0	98.7	€ 678	€ 19	€ 596	€ 630	€ 0
*2016*	100	€ 861	€ 50	€ 474	€ 650	€ 0	98.7	€ 723	€ 68	€ 526	€ 772	€ 0
*2017*	98.8	€ 442	€ 37	€ 354	€ 415	€ 0	98.1	€ 770	€ 70	€ 640	€ 769	€ 1,654
*2018*	98.2	€ 419	€ 48	€ 358	€ 470	€ 0	97.6	€ 798	€ 85	€ 662	€ 778	€ 1,813
*2019*	97.2	€ 494	€ 56	€ 449	€ 539	€ 0	97.3	€ 936	€ 95	€ 650	€ 807	€ 1,785

^a^*Total* all medication

^b^*Oral* oral blood glucose lowering medication (A10B); *Insulin* insulin (*A10A*); *Oral/insulin* a combination of oral blood glucose lowering medication; *Other* combination medication (A10AE54 or A10AE56)

**Fig. 2:**
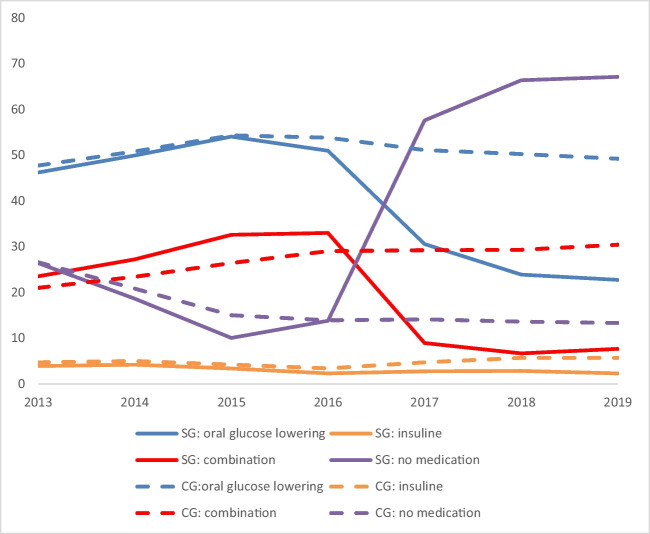
Overview of percentages of patients in the surgery group (SG) and control group (CG) using medication for diabetes, divided in four groups: (1) oral blood glucose lowering medication (A10B); (2) insulin (*A10A*); (3) a combination of oral blood glucose lowering medication or a combination medication (A10AE54 or A10AE56); and (4) no medication

In the surgery group, there was a decrease in median expenditures for oral blood glucose lowering medication after 2016, insulin and combination treatment after 2016 (Table 3, Fig. 2 and supplemental Table 1). Also, for utilisation of diabetes medication, there was an increase up to the surgery year and then a decrease until 2019: 67.1% did not use any glucose-lowering medication in 2019, compared to 13.8% in 2016. For the control group, there were increases in all expenditures and utilisation, and the percentage of patients not using medication decreased to 13% in 2019.

**Fig. 3:**
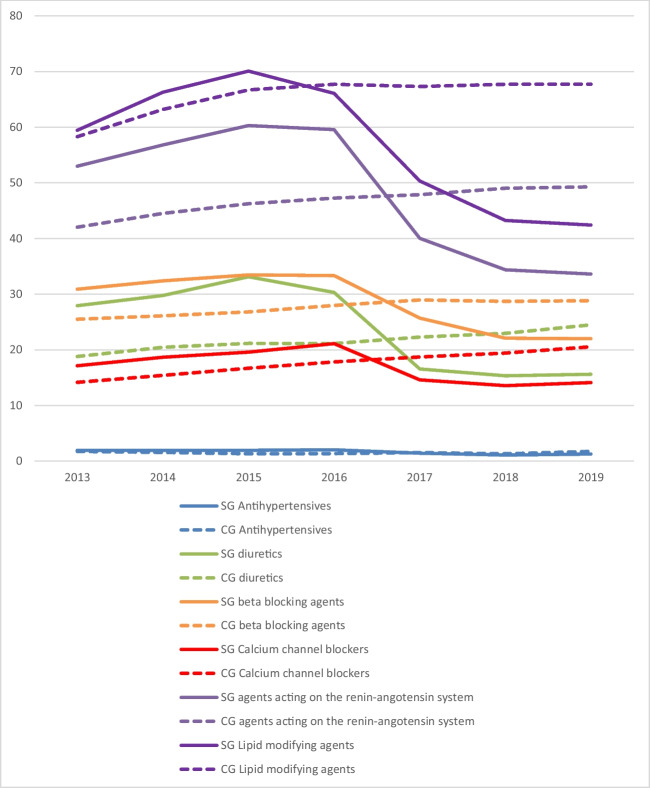
Overview of percentages of patients in the surgery group (SG) and control group (CG) using medication for associated medical conditions of type 2 diabetes

There was also a decrease in use of diuretics, beta-blocking agents, calcium channel blockers and agents acting on the renin-angiotensin system after BMS (Fig. 3 and supplemental Table 2). The largest change was in agents acting on the renin-angiotensin system (C09), with a decrease from 53.0 to 33.6% of the people using this medication after surgery. In the control group, utilisation of all these medications increased over all study years.

### Diabetes-Related Complications

In Table 4, healthcare utilisation and expenditures for vascular complications associated with T2DM are shown. In the surgery group, the utilisation of care for microvascular complications was highest in 2016 (10.4%) and then decreased to 9.7% in 2019. In the control group, utilisation for microvascular complications increased to 13.2% in 2019. Healthcare utilisation for macrovascular complications was stable in both groups, but always higher in the control group.

**Table 4:** Annual care utilisation (% of group) and median expenditures (p5 and p95) per patient for micro- and macrovascular complications, for the surgery and control group

*Year*	Surgery group (1751)								Control group (*n*=3502)							
	Microvascular				Macrovascular				Microvascular				Macrovascular			
	Utilisation	Expenditures			Utilisation	Expenditures			Utilisation	Expenditures			Utilisation	Expenditures		
		Median	P5	P95		Median	P5	P95		Median	P5	P95		Median	P5	P95
*2013*	7.8	€ 183	€ 88	€ 9407	2.5	€ 2343	€ 564	€ 15,677	8.8	€ 275	€ 88	€ 7862	3.8	€ 1839	€ 197	€ 28,678
*2014*	8.3	€ 364	€ 89	€ 3435	3.5	€ 1823	€ 364	€ 24,864	9.3	€ 318	€ 89	€ 7858	3.7	€ 2526	€ 196	€ 19,384
*2015*	7.7	€ 238	€ 87	€ 4947	2.7	€ 1815	€ 211	€ 12,752	9.6	€ 355	€ 87	€ 4859	2.9	€ 1459	€ 185	€ 23,551
*2016*	10.4	€ 283	€ 87	€ 4020	2.8	€ 721	€ 208	€ 12,961	11.4	€ 283	€ 83	€ 7147	3.1	€ 1126	€ 208	€ 22,349
*2017*	10.3	€ 259	€ 80	€ 4888	2.8	€ 673	€ 200	€ 29,035	11.8	€ 282	€ 85	€ 8416	3.5	€ 1146	€ 200	€ 12,678
*2018*	9.7	€ 689	€ 87	€ 4866	2.5	€ 945	€ 200	€ 10,326	12.4	€ 400	€ 83	€ 7820	3.3	€ 1594	€ 200	€ 22,952
*2019*	9.7	€ 547	€ 87	€ 9665	2.5	€ 867	€ 453	€ 12,761	13.2	€ 449	€ 84	€ 8816	3.6	€ 1910	€ 203	€ 23,170

Expenditures for microvascular complications increased in both groups. For macrovascular complications, there was a decline in the surgery group from €2342 in 2013 to €867 in 2019, but not in the control group.

## Discussion

The goal of this study was to explore changes in healthcare expenditures and utilisation following BMS in people with T2DM, using real-world data from a national APCD that covers all Dutch citizens. Among persons that underwent BMS, total healthcare expenditures increased up to the surgery year and after that decreased to below the level of expenditures at study start in 2013. While in the group that did not undergo surgery, expenditures continued to rise over time. Three years after surgery, only one-third of people in the surgical group was using glucose lowering medication, and there was a substantial decline in use of medication for diabetes associated medical conditions (i.e. hypertension and dyslipidaemia) possibly indicating remission of T2DM and associated morbidity. Total pharmaceutical expenditures decreased with 28% in the surgical group, compared to a rise of 50% in the control group. Furthermore, in the control group, healthcare utilisation for microvascular complications of T2DM increased over time, while there was a decrease in the surgery group.

Median healthcare expenditures per patient in the surgery group decreased at the end of the study (2019) to median costs about 1.1% below the costs in 2013. It seems counterintuitive that this study did not find a large decline in healthcare expenditures when comparing before and after surgery, since several reviews have concluded that BMS is cost-effective for people with T2DM [14, 15]. However, it is crucial to differentiate between cost-effectiveness (i.e. ratio of costs and effect) and cost-saving (i.e. comparing cost before and after surgery). Only one previous study assessed whether BMS is cost-saving on the short term (2 years) in people with T2DM; they also did not find a large decrease in costs after surgery [16]. A study including all people with obesity with a 6-year follow-up after surgery also concluded that surgery was not cost-saving on the short term [19]. This is probably caused by the high costs of the surgical procedure itself, which up to $30,000/€27,000 [11, 12, 16, 19].

Moreover, it is important to note that expenditures for complications are the most expensive part of healthcare for people with T2DM which continue to increase over time [18]. In the current study, healthcare expenditures and utilisation of the surgery group stabilised or improved slightly, whereas these improvements were not observed for the control group. For instance, there was not a large decline in expenditures in the first 3 years after surgery, but there was an increase in expenditures in the group that was not treated with BMS. For this group, expenditures were highest in the last study year: median costs were € 3434 in 2019, which was almost a 7% increase compared to 2018. These rising costs were mostly attributable to more medication use and more utilisation of primary and secondary care. Additionally, 3 years after surgery, only one-third of the population who had surgery used medication for diabetes, while this was almost 90% in the group who did not undergo surgery. This resulted in pharmaceutical expenditures in the surgical group of almost half of the expenditures in non-surgical group. More importantly, this indicates the surgery group experienced metabolic benefits of BMS which are shown to positively affect severity and progression of T2DM [5–10, 20].

The progression of T2DM often results in more use of medication and micro- and macrovascular complications. Even with the relative short-term follow-up of 3 years, our data already showed that the percentage of people utilizing healthcare for microvascular complications of T2DM continued to increase in the control group and not in the surgical group. Prior research in diabetes patients shows similar benefits of BMS in decreasing microvascular complications [21] as well as cardiovascular risk [22]. As T2DM is a progressive disease, it is expected that this rise in healthcare expenditures and utilisation in the people who do not undergo BMS will continue to increase over time, which is probably the reason that surgery is cost-saving in patients with T2DM over a time horizon longer than 10 years [15].

The current study also allows us to assess which patients with T2DM undergo BMS in the Netherlands. It shows that a small percentage of people with T2DM is treated with BMS (0.19%; 1700 of the 880,121 people with T2DM in 2016) [18], while up to 22.5% of Dutch persons with T2DM have obesity [23] and BMS has proven health benefits for this group [4–10]. One possible explanation of this gap could be age-related: in the current study, mean age was 52 years; in the total diabetes population, this was 67 years, which was previously considered too old for BMS [18, 24]. Also, compared to the whole T2DM population in the Netherlands, the rates of micro- and macrovascular complications were lower in our study sample, as were medication costs [18, 25]. In accordance to previous research, younger people with less advanced T2DM are treated with BMS in the Netherlands [26]. This raises the question whether the population of people with T2DM who would benefit most from BMS is also treated with surgery. Though results in terms of glycaemic control are better in people with less severe T2DM, people with more progressed disease would likely benefit from BMS as well [26].

A limitation of this study is that there was no clinical data (e.g. body weight, HbA1c or glucose levels) available for the matching procedure of surgery and control group. Hence, to ensure that people in the study groups were comparable in terms of health, we used total healthcare expenditures in the previous years (2013–2014), as a proxy for general health status. However, the lack of detailed clinical data and the retrospective nature of the study may have led to some selection bias, as we are unsure whether people with T2DM in the control group have obesity (BMI≥30kg/m^2^). We expect that this bias would have resulted in lower utilisation and expenditures in control group and thus an underestimation of the difference between the groups. It is important to remember that in previous research, the benefits of surgery for people with diabetes already start at a BMI of 27 kg/m^2^ [26, 27]. Notwithstanding this limitation, this is the first nationwide analyses of changes in healthcare expenditures and utilisation in people with T2DM who undergo BMS. The database used for this study contains data of almost all Dutch citizens (99% coverage). Based on publications of the mandatory nationwide registry for BMS in the Netherlands, Dutch Audit for Treatment of Obesity, around 85% of the people with T2DM who had surgery in 2016 were included in the current study [28, 29]. This difference can be caused by the fact that reimbursement claims are sometimes delayed, resulting in a claim in the next year.

This data shows to be very promising for further analyses. Using the current database for future research would mean no loss to follow-up and thus also show the exact extent of changes in healthcare utilisation and expenditures. However, potential benefits from BMS are not limited to healthcare utilisation and expenditures alone. Prior research has shown benefits of BMS in T2DM patients regarding clinical measures and quality of life [5–10, 20, 30]. The current study based on claims data points to similar benefits: medication rates and healthcare use of the surgery group declined after BMS, likely associated with improved health and quality of life. Therefore, it would be meaningful to shift to a more holistic view and assess both clinical factors and quality of life for the nationwide APCD cohort in future research. Such data could also be used to study which people with T2DM benefit most from BMS.

In conclusion, in people with T2DM, BMS leads to stabilisation/decline in healthcare expenditures, while over the same time period, expenditures rise in people with T2DM who are not treated with surgery. Due to the progressive nature of T2DM, it is expected that these differences will be more apparent on the long term after surgery. Moreover, only a fraction of the people with T2DM in the Netherlands is treated with BMS. Combining reimbursement data with clinical data will allow for more insight in improvement of selection criteria for surgery.

### Supplementary Information

ESM 1 (DOCX 14 kb)

## Data Availability

The data sets generated and analysed during this study are not publicly available. Data are, however, available from Vektis upon reasonable request and with formal consent of the Dutch health insurers.
