# Supplementary material for: Evaluation of Healthcare Utilisation and Expenditures in Persons with Type 2 Diabetes Undergoing Bariatric-Metabolic Surgery
Source: Obes Surg. 2024 Jan 10;34(3):723–32. doi: 10.1007/s11695-023-06849-z (PMC10899363; doi:10.1007/s11695-023-06849-z)
Supplement: Supplementary file 1 — (DOCX 14 kb) [file 11695_2023_6849_MOESM1_ESM.docx]

| **Supplemental table 1:**  **Percentage of patients per year using medication for type 2 diabetes, for the surgery group and control group.** | | | | | | | | |
| --- | --- | --- | --- | --- | --- | --- | --- | --- |
|  | **Surgery group**  **(1,751)** | | | | **Control group**  **(n=3,502)** | | | |
| *Year* | **Oral** | **Insulin** | **Oral/**  **insulin** | **No medication** | **Oral** | **Insulin** | **Oral/**  **insulin** | **No medication** |
| *2013* | 46 | 4 | 24 | 26 | 48 | 5 | 21 | 27 |
| *2014* | 50 | 4 | 27 | 19 | 51 | 5 | 23 | 21 |
| *2015* | 54 | 3 | 33 | 10 | 54 | 4 | 26 | 15 |
| *2016* | 51 | 2 | 33 | 14 | 54 | 3 | 29 | 14 |
| *2017* | 31 | 3 | 9 | 58 | 51 | 5 | 29 | 14 |
| *2018* | 24 | 3 | 7 | 66 | 50 | 6 | 29 | 14 |
| *2019* | 23 | 2 | 8 | 67 | 49 | 6 | 30 | 13 |
| Oral: oral blood glucose lowering medication (A10B); Insulin: insulin (A10A); Oral/insulin: a combination of oral blood glucose lowering medication or combination medication (A10AE54 or A10AE56); | | | | | | | | |

| **Supplemental table 2:**  **Percentage of patients per year using medication for associated medical conditions of type 2 diabetes, for the surgery group and control group.** | | | | | | | | |
| --- | --- | --- | --- | --- | --- | --- | --- | --- |
|  |  | **2013** | **2014** | **2015** | **2016** | **2017** | **2018** | **2019** |
| Surgery group | Antihypertensives | 2 | 2 | 2 | 2 | 1 | 1 | 1 |
|  | Diuretics | 28 | 30 | 33 | 30 | 17 | 15 | 16 |
|  | Beta blocking agents | 31 | 32 | 33 | 33 | 26 | 22 | 22 |
|  | Calcium channel blockers | 17 | 19 | 20 | 21 | 15 | 14 | 14 |
|  | Agents acting on the renin-angotensin system | 53 | 57 | 60 | 60 | 40 | 34 | 34 |
|  | Lipid modifying agents | 59 | 66 | 70 | 66 | 50 | 43 | 42 |
| Control group | Antihypertensives | 2 | 2 | 1 | 1 | 2 | 1 | 2 |
|  | Diuretics | 19 | 20 | 21 | 21 | 22 | 23 | 24 |
|  | Beta blocking agents | 25 | 26 | 27 | 28 | 29 | 29 | 29 |
|  | Calcium channel blockers | 14 | 15 | 17 | 18 | 19 | 19 | 21 |
|  | Agents acting on the renin-angotensin system | 42 | 45 | 46 | 47 | 48 | 49 | 49 |
|  | Lipid modifying agents | 58 | 63 | 67 | 68 | 67 | 68 | 68 |
